# The actinin family of actin cross-linking proteins – a genetic perspective

**DOI:** 10.1186/s13578-015-0029-7

**Published:** 2015-08-25

**Authors:** Anita C.H. Murphy, Paul W. Young

**Affiliations:** School of Biochemistry and Cell Biology, University College Cork, Cork, Ireland

**Keywords:** Actinin, Alpha-actinin, ACTN1, ACTN2, ACTN3, ACTN4, Actin cross-linking, Actin binding proteins

## Abstract

Actinins are one of the major actin cross-linking proteins found in virtually all cell types and are the ancestral proteins of a larger family that includes spectrin, dystrophin and utrophin. Invertebrates have a single actinin-encoding *ACTN* gene, while mammals have four. Mutations in all four human genes have now been linked to heritable diseases or traits. *ACTN1* mutations cause macrothrombocytopenia, a platelet disorder characterized by excessive bleeding. *ACTN2* mutations have been linked to a range of cardiomyopathies, and *ACTN4* mutations cause a kidney condition called focal segmental glomerulosclerosis. Intriguingly, approximately 16 % of people worldwide are homozygous for a nonsense mutation in *ACTN3* that abolishes actinin-3 protein expression. This *ACTN3* null allele has undergone recent positive selection in specific human populations, which may be linked to improved endurance and adaptation to colder climates. In this review we discuss the human genetics of the *ACTN* gene family, as well as *ACTN* gene knockout studies in several model organisms. Observations from both of these areas provide insights into the evolution and cellular functions of actinins.

**Electronic supplementary material:**

The online version of this article (doi:10.1186/s13578-015-0029-7) contains supplementary material, which is available to authorized users.

## Introduction

**Fig. 1:**
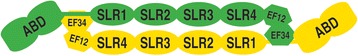
Schematic representation of the actinin dimer. The domain organization of the anti-parallel actinin dimer is depicted schematically in the closed conformation as observed in the X-ray crystallographic structure of the human actinin-2 [4]. In each subunit two calponin homology (CH) domains make up the N-terminal actin-binding domain (ABD). The rod domain composed of four spectrin-like repeats (SLR1-4) makes up the bulk of the dimer interface. The carboxyl terminal calmodulin-like (CaM) domain is made up of two pairs of EF-hand motifs (EF1/2 and EF3/4). EF1/2 binds Ca^++^ in some actinins [1]. EF3/4 from one subunit interacts with the “neck” region between the ABD and first SLR of the opposing monomer (depicted as a line) [3]. This interaction clamps the protein into a closed conformation that is thought to be opened up by phospholipid binding to the ABD [4, 3]

Actinins are dimeric actin filament cross-linking proteins. At their amino terminus two adjacent calponin homology (CH) domains comprise an actin-binding domain (ABD) [1]. Anti-parallel dimerisation of actinin monomers, mediated by the central spectrin-like repeats (SLRs) [2], positions the actin-binding domains at either end of the rod shaped dimer and facilitates cross-linking of actin filaments (Fig. 1). The carboxyl terminal calmodulin-like (CaM) domain, made up of two pairs of EF-hand motifs (EF1/2 and EF3/4), plays a regulatory role. EF1/2 can bind Ca^++^ in some actinins, disrupting actin binding at high Ca^++^ concentrations [1]. A postulated interaction of EF3/4 with the “neck” region between the ABD and first SLR of the opposing monomer [3] has been confirmed by the recently determined X-ray crystallographic structure of the human actinin-2 dimer [4]. This structure reveals actinin to be in a “closed” conformation. Phospholipid binding to the ABD causes a conformational change, resulting in the “opening” of the structure that allows EF3/4 to bind the sarcomeric organizer protein titin [5, 6] – a potential mechanism to regulate titin and actinin integration into the muscle Z-disk. More broadly though, this structure presents a picture of the intimate interactions between the subunits in the actinin dimer, providing a framework to better understand the molecular details of actin cross-linking and its regulation in all actinins and perhaps in spectrins too.

Actinins would appear to have first evolved in a common ancestor of amoebas, fungi and yeast [7, 8]. Thus, they were a component of early eukaryotic actin-based cytoskeletons, though notably, they are not found in plants. Actinin is also thought to be the ancestor of the spectrin and dystrophin families of actin-binding proteins, with spectrins probably evolving in the immediate ancestors of metazoans, based on their presence in choanoflagellates [9, 10], and the dystrophins arising in early metazoans [11]. As eukaryotic cells evolved, cross-linking of microfilaments, and anchoring them to membranes and other subcellular structures, were probably key to effective actin-based force generation, whether by myosin, or through actin polymerization [12]. In particular, actinins seem to have collaborated successfully with myosin II motors to assemble the actin-based contractile systems required for diverse cellular processes, such as cytokinesis, cell motility and muscle contraction. Reflecting these fundamental functions, the basic domain organization of actinins has remained unchanged through evolution, with the exception of some variability in the number of SLRs (two in most fungal actinins versus four in virtually all other taxa) [8]. A multiple sequence alignment of the actinin proteins discussed in this review is provided as additional information [Additional file 1]. Invertebrates generally have a single *ACTN* gene, while vertebrates usually have three (or more), presumably as a result of the two rounds of large-scale gene, or whole-genome, duplication that are thought to have occurred early in the vertebrate lineage (2R hypothesis) [13]. Vertebrate actinins can be classified as either Ca^++^- sensitive or -insensitive with respect to actin binding. This arises from alternative splicing of two variants of exon 19, which encodes part of the first EF-hand motif. Alternative splicing of this exon seems to have arisen in chordates [14]. The exon 19a and 19b variants can generate Ca^++^- sensitive and -insensitive actinin isoforms respectively. Of the four mammalian actinins, alternative splicing of exon 19 has been retained in *ACTN1* and *ACTN4* to generate Ca^++^-sensitive variants that are widely-expressed, and Ca^++^-insensitive variants that are found predominantly in smooth muscle and the central nervous system (CNS) [1, 15]. In contrast, *ACTN2* and *ACTN3* exclusively encode exon 19b-containing Ca^++^-insensitive proteins that are expressed in electrically excitable cells (cardiac and skeletal muscle cells, as well as neurons) [16, 17].

Here we review genetic studies of actinin function, summarizing findings both from model organisms (ranging from yeast to mice) and from human disease-association studies. This survey highlights the multiple functions of this versatile actin cross-linker, some of which are evolutionarily conserved, and others that probably represent specialized roles acquired in particular phyla or species.

### Genetic studies of actinins in non-vertebrate model organisms

#### Yeast

Fission yeast (*Schizosaccharomyces pombe*), but not budding yeast (*Saccharomyces cerevisiae*), possess an actinin protein. It has only two spectrin-like repeats and is thought to be Ca^++^-insensitive with respect to actin binding [18]. *S. pombe* actinin has mainly been studied with regard to contractile ring assembly during cytokinesis. The contractile ring forms from complexes termed nodes in the middle of the cell during interphase. Actinin is not essential for cytokinesis under normal growth conditions, but actinin null yeast show severe cytokinesis defects under stressful conditions of low temperature and high salt [19]. While actinin null cells do complete cytokinesis under normal growth conditions, the formation of the contractile ring is delayed [20]. This is because the actin network is more dynamic during node condensation, and the cytokinesis nodes frequently condense into clumps before eventually recovering to form a contractile ring. In cells over expressing actinin, actin filament structures are stabilized, inhibiting myosin-dependent movements of nodes that are essential for node condensation [20]. This results in a delayed ring assembly, but for reasons different to those in actinin null cells. Not surprisingly, there is considerable redundancy in the machinery of cytokinesis. Fimbrin is another actin cross-linker involved in this process. It seems to be able to compensate to a large extent for loss of actinin (and vice versa), since cells null for both these proteins have more severe defects in contractile ring formation than single mutants [20, 19]. Overall, it appears that in yeast, a balance between actin cross-linking and myosin motor activity must be achieved in order for cytokinesis to proceed in a controlled fashion – a concept that seems to hold true in mammalian cells too [21].

#### Slime molds

The mycetozoan *Dictyostelium discoideum* is an excellent unicellular model in which to study the role of the cytoskeleton in cell biological processes that are shared with multi-cellular animals. Actinin null *D. discoideum* strains are largely normal, but exhibit slow growth under hyper-osmotic conditions [22]. However, much more severe defects are seen in double mutants lacking actinin and either of the actin-bundling proteins filamin (ABP120, gelation factor) or ABP34. These double mutants show decreased growth at normal osmolarity, deficient pinocytosis, motility defects and abnormal morphogenesis of the multi-cellular fruiting body structure [22]. A study which employed more natural growth conditions revealed further deficiencies in the ability of actinin single null mutants to efficiently complete development and form viable spores [23]. These observations point to important roles for actinin in several dynamic processes in *D. discoideum,* but with significant though not complete, functional redundancy between actinin and other actin cross-linking proteins – a similar conclusion to the studies in fission yeast.

#### Flies and worms

*Drosophila melanogaster* has a single *ACTN* gene that is alternatively spliced within the region encoding the ABD. This alternative splicing generates isoforms that are differentially expressed in non-muscle, larval muscle and adult muscle tissues [24]. Embryos harboring null *ACTN* alleles that eliminate all isoforms can complete embryogenesis, but larvae die within two days of hatching [25]. These larvae are capable of some degree of movement, but have severely disrupted myofibrils, show muscular degeneration, and experience paralysis that is ultimately lethal [26]. Nevertheless, the initial stages of sarcomere assembly do not seem to require actinin, an observation confirmed in more recent studies using RNA interference targeting actinin [27, 28]. These studies suggest that in flies, actinin functions to stabilize the forming sarcomeric Z-disk, rather than initiating its assembly. In addition, other non-muscle aspects of embryogenesis in actinin null *D. melanogaster* seemed to proceed normally in the absence of actinin [24], and in mosaic flies, in which patches of eye tissue lacked actinin, the morphology of ommatidial rhabdomeres was normal [26].

Studies in *Caenorhabditis elegans* yield quite similar conclusions to those in flies. Worms harboring a deletion of the sole *C. elegans ACTN* gene (*ATN-1*) exhibit structural abnormalities of the focal adhesion-like dense bodies in the body wall muscle, though the dense bodies still accumulate integrin, talin and vinculin [29]. Muscle cells exhibit actin striations, but with some actin disorganization and abnormal accumulations of actin in bundles at cell boundaries. Nevertheless, the mutant worms show nearly normal movement in several assays, though a deficiency in the amplitude of body bending was discernible by video-microscopy [29]. Similar to flies, non-muscle aspects of development seem to proceed normally in actinin null *C. elegans*, suggesting that actinin is not absolutely required for many fundamental processes, such as cytokinesis, cell-cell adhesion or cell motility. The apparent absence of non-muscle phenotypes in flies and worms suggests that either actinin does not play an important role in such processes in these species, or that its absence can be compensated for to a significant extent by other proteins.

### Genetic studies of actinins in mammals

Mammals have four ACTN genes. *ACTN1* and *ACTN4*, through alternative splicing, encode widely-expressed Ca^++^-sensitive isoforms, as well as Ca^++^-insensitive variants that are found predominantly in smooth muscle and in the central nervous system (CNS) [30]. *ACTN2* and *ACTN3* encode Ca^++^-insensitive proteins only, expressed predominantly in cardiac and skeletal muscle, but also in the CNS in the case of *ACTN2* [16, 17]. Mutations in all four human ACTN genes have now been linked to heritable diseases or traits. In addition, knockout mice lacking actinin-3 and actinin-4 have been described [31, 32]. Genetic studies of each mammalian actinin are discussed in turn below.

#### Actinin-1

In many cell types, actinin-1 cross-links actin filaments and anchors them to structures such as cell:cell and cell:matrix junctions, playing an important role in processes such as cytokinesis, cell adhesion and cell migration [33]. The International Mouse Phenotyping Consortium has performed initial phenotyping of mice heterozygous for an *ACTN1* knockout allele [34]. Presumably heterozygotes were used because homozygous mice were not viable, though there is little background information available about these mice. In any case, it would not be surprising if *ACTN1* were an essential gene, given its ubiquitous expression and many described functions.

A role for actinin-1 in a human genetic disorder has become apparent in the past two years, with three independent studies showing *ACTN1* to be one of many causative genes implicated in dominantly-inherited congenital macrothrombocytopenia (CMTP) [35–37]. This is a rare blood disorder characterized by a reduced number of platelets in the peripheral vascular system along with increased platelet size [38]. Initially, Kunishima et al. [37] identified six *ACTN1* variants that co-segregated with affected individuals in six Japanese families suffering with CMTP. Expression of these variants in Chinese hamster ovary cells and primary mouse fetal liver-derived megakaryocytes lead to abnormal alterations in actin cytoskeleton organization [37]. Gueguen et al. [36] subsequently reported an ACTN 1 variant, Arg46Gln, to be co-segregating with CMTP in a French family. This missense mutation had already been reported by Kunishima et al., but its appearance in an independent patient cohort supplies reliable evidence for the definite involvement of *ACTN1* in CMTP. Most recently, Bottega et al. reported 8 new *ACTN1* variants in families from Italy and the United Kingdom [35]. Expression of corresponding mutant actinin-1 proteins in human fibroblast cultured cells caused actin cytoskeleton disorganization. It has been suggested that a possible cause for macrothrombocytopenias is a deficiency in the regulation of platelet production [39]. In support of this, primary mouse fetal liver-derived megakaryocytes transfected with *ACTN1* variants displayed altered pro-platelet formation and size [37]. This finding is compatible with the increased platelet size that is characteristic of CMTP.

Intriguingly, all of the actinin-1 mutations linked to CMTP map within the ABD and CaM domains, or to regions that link these domains to the central rod domain (Table 1). No CMTP-associated mutations have been found within the rod domain itself, even though the rod encompasses half of the actinin protein sequence. This observation, together with the cytoskeletal abnormalities seen upon heterologous expression, suggests that these actinin-1 variants may have altered actin-binding properties. However, this has not been directly examined to date, and the location of these mutations on the three-dimensional structure of the actinin dimer does not provide obvious clues as to how they might affect actin-binding properties [4]. Individuals heterozygous for these mutations display relatively mild macrothrombocytopenia, apparently in the absence of other pathologies. This finding points to a specific role for actinin-1 in platelet formation, possibly through its actin binding and bundling ability, that is exquisitely sensitive to mutational perturbation and cannot be compensated for by other actinin isoforms.

**Table 1:** Human actinin mutations and associated diseases or phenotypes

Protein	Mutation	Domain	Disease/Phenotype	Reference
Actinin-1	Asp22Asn	ABD	CMTP	[35]
	Gln32Lys	ABD, CH1	CMTP	[37]
	Arg46Gln	ABD, CH1	CMTP	[36, 37]
	Arg46Trp	ABD, CH1	CMTP	[35]
	Val105Ile	ABD, CH1	CMTP	[37]
	Glu225Lys	ABD, CH2	CMTP	[37]
	Gly251Arg	ABD/Neck	CMTP	[35]
	Thr737Asn	Rod/CaM	CMTP	[35]
	Arg738Trp	Rod/CaM	CMTP	[37]
	*Arg752Gln	CaM, EF12	CMTP	[37]
	Gly764Ser	CaM, EF12	CMTP	[35]
	Glu769Lys	CaM, EF12	CMTP	[35]
Actinin-2	Gln9Arg	ABD	DCM	[41]
	Gly111Val	ABD, CH1	HCM	[42]
	Ala119Thr	ABD, CH1	HCM, DCM, IVF, LVNC, SUD	[45, 44]
	Met228Thr	ABD, CH2	HCM + Atrial Arrhythmias	[46]
	Thr495Met	Rod, SLR2	HCM	[44, 42]
	Glu583Ala	Rod, SLR3	HCM	[44]
	Glu628Gly	Rod, SLR3	HCM	[44]
	*Arg759Thr	CaM, EF12	HCM	[42]
Actinin-3	Arg577X	Rod, SLR3	Altered muscle metabolism	[50]
Actinin-4	Trp59Arg	ABD, CH1	FSGS	[77]
	Ile149del	ABD, CH1	FSGS	[77]
	Lys255Glu	ABD, CH2	FSGS	[76]
	Thr259Ile	ABD, CH2	FSGS	[76]
	Ser262Pro	ABD, CH2	FSGS	[76]

Mutations in the four human actinin proteins that are associated with diseases or phenotypic traits are listed.

The protein domain and subdomain (e.g. ABD, CH1) in which each mutation occurs is indicated in the third column. A forward slash (e.g. Rod/CaM) indicates a mutation within a region linking the two domains indicated. The asterisk indicates that these are equivalent Arg residues in a sequence alignment of actinin-1 and −2. A multiple sequence alignment highlighting the postions of disease-causing mutations is provided as additional information [Additional file 1].

*Abbreviations*: *IVF* Idiopathic ventricular fibrillation, *LVNC* Left ventricular non-compaction, *SUD* Sudden unexplained death, *CMTP* Congenital macrothrombocytopenia, *HCM* Hypertrophic cardiomyopathy, *DCM* Dilated cardiomyopathy, *FSGS* Focal segmental glomerulosclerosis

#### Actinin 2

Actinin-2 is expressed in skeletal and cardiac muscle fibers [16], as well as in the brain [17]. In muscle, it is a highly abundant protein and is the major Z-disk protein that cross-links anti-parallel actin filaments from neighboring sacromeres. Actinin-2 also serves as a scaffold to which many other Z-disk components are anchored, and may be involved in mechanical strain sensing and signaling through interacting proteins such as CSRP3/MLP, PDLIM3/ALP and LDB3/ZASP/cypher [40]. A number of investigations have now linked dominantly-inherited *ACTN2* missense mutations to a range of myopathies. An *ACTN2* mutation was reported in a patient with dilated cardiomyopathy (DCM), a condition characterized by dilation of the left ventricle of the heart and a reduction in the heart’s ability to contract [41]. This Gln9Arg mutation is found in the ABD of actinin-2 and abrogates an interaction of actinin-2 with the Z-disk component MLP [41]. Examining patients with hypertrophic cardiomyopathy (HCM), Theis et al. identified thirteen mutations in five Z-disk proteins, including three in actinin-2 [42]. HCM is a chronic heart muscle disease that affects about 0.2 % of people, and is characterized by a thickened wall of the left ventricle in the heart [43]. Semsarian and coworkers found one of these same mutations (Thr495Met), as well as three novel mutations in Australian families affected by HCM and other heterogeneous cardiac conditions [44, 45]. Similarly an actinin-2 Met228Thr mutation was found to segregate with affected individuals in a large Italian family that had a history of HCM and juvenile atrial arrhythmias [46]. Eight *ACTN2* mutations have thus far been linked to HCM, DCM and/or other cardiac abnormalities. These mutations do not map to a particular region of actinin-2, with some located in the ABD, some in the central rod and one in the CaM domain (Table 1). While mutations that map to the ABD and CaM domains may affect actin-binding properties, those in the rod domain are more likely to affect the binding of other Z-disc proteins to α-actinin. However the consequences of these mutations have not been examined experimentally for the most part. Given the distribution of the mutations, it would appear unlikely that there is a unifying molecular mechanism linking them. In some of the studies, individuals from a family carrying the same *ACTN2* mutation experience profound clinical and phenotypic heterogeneity [44]. For example the Ala119Thr substitution was identified in a family with a history of DCM, left ventricular non-compaction, idiopathic ventricular fibrillation and unexplained sudden death [44]. This suggests that factors other than genetics, such as diet, exercise and environmental conditions, are also likely to influence clinical outcome [47]. The availability of a high resolution actinin-2 crystal structure will be useful to further our understanding of how these mutations affect actinin-2 function and ultimately cause disease [4].

### Actinin 3

Actinin-3 seems to be the most specialized of the mammalian actinins based on its restricted expression in type 2 fast glycolytic skeletal muscle fibers, the fibers responsible for the generation of rapid and forceful contractions [16, 48, 49]. Surprisingly, an *ACTN3* polymorphism, causing the nonsense mutation p.Arg577X, was found to be very prevalent in many human populations [50]. Approximately 16 % of the world’s population are homozygous for this sequence change that completely prevents the production of the actinin 3 protein, meaning that more than one billion people lack actinin-3 expression [51]. This null genotype is not associated with any disease, suggesting that *ACTN3* is a non-essential gene in humans and its loss compensated for by actinin-2 [50]. Though absent in birds, the *ACTN3* gene is conserved in most other vertebrates, including fish, suggesting that it arose by gene duplication early in vertebrate evolution [52]. Actinin-3 must have had non-redundant functions through the course of vertebrate evolution in most lineages to explain its sequence conservation. In early humans however, it appears that the p.Arg577X mutation arose, was not detrimental, and was maintained for some time, before expanding under positive selection to achieve a very high frequency in specific populations (e.g. European and Asian), but not others (e.g. African) [49]. What is the basis for this highly unusual, positive selection for a null allele?

Yang et al. [53] initially reported an overrepresentation of the wild type *ACTN3* allele (p.Arg577Arg) in elite Australian sprint athletes, suggesting that its presence is advantageous in sprint and power activities. They also found the homozygous p.Arg577X genotype to be more common in female endurance athletes when compared to power athletes. This suggested that *ACTN3* genotype is linked to normal variation in muscle function, with each genotype possibly conferring an advantage for different types of athletic performance. Numerous other studies have subsequently investigated the association between *ACTN3* genotype and athletic performance, in both normal populations and various groups of elite athletes. Some studies support the basic findings of Yang et al. [53], while others do not find significant associations. A complete discussion of this literature is beyond the scope of this review but is covered quite comprehensively by Eynon et al. [54]. However, it can be concluded that at least in some populations (e.g. Caucasians), the association of the wildtype allele with sprint and power performance seems to hold true, while the association of the p.Arg577X variant with enhanced endurance is not as clear cut [54, 55]. Notably though, in African populations, where the frequency of the p.Arg577X variant is very low, no association of *ACTN* genotype with elite athlete status was found [56], despite Kenyans and Ethiopians having dominated long–distance running in recent years [57]. This suggests that we must consider not only the *ACTN3* genotype but also other polymorphisms that may be working in combination with, or independently of, *ACTN3* to dictate athletic performance [55]. In addition, athletic performance depends not just on an individual’s genetic make-up, but also on environmental factors, training regimes and coaching expertise [54, 55].

Studies of *ACTN3* knockout mice provide further insights [58]. These mice are viable and healthy. They do exhibit slight decreases in muscle mass and muscle strength, but these values are regarded as within the normal range and are not a sign of muscle dysfunction [59]. More significantly, these mice display muscle metabolism conversion from the anaerobic pathway, typically utilized in fast muscle fibers, to the oxidative aerobic pathway, which is generally seen in slow muscle fibers [58]. Activity of key enzymes associated with oxidative metabolism, such as citrate synthase and succinate dehydrogenase, and glycolysis, such as hexokinase, are increased, while indicators of anaerobic metabolism, namely glycogen phosphorylase, show decreased activity. These metabolic changes have a positive effect on endurance; knockout mice have a much greater running distance prior to experiencing fatigue compared to wild-type controls [58]. The function of glycogen phosphorylase is to break down glycogen. In humans, activities such as sprinting rely on glycogen as a main source of energy, a reduction in glycogen breakdown would therefore be unfavorable to sprint athletes [59]. However, a reduced ability to breakdown glycogen might be beneficial to endurance athletes, as it allows them to utilize other fuels and conserve glycogen [60]. Thus, metabolic changes observed in *ACTN3* knockout mice provide plausible explanations for the association of *ACTN3* genotypes with sprint/power versus endurance performance in humans. More efficient aerobic muscle metabolism may be the trait associated with the p.Arg577X genotype that has been positively selected for in specific human populations. The frequency of this allele in human ethnic groups is correlated with latitude, with the p.Arg577X mutation more prevalent further from the equator [61]. Latitude-associated environmental variables, such as mean temperature or species diversity, may have influenced positive selection for this *ACTN3* allele [61]. Indeed, recently described alterations in the calcium kinetics in skeletal muscles of *actinin-3* knockout mice are consistent with cold acclimatization and thermogenesis [62, 60].

What molecular mechanisms might mediate these powerful effects of actinin-3 on multiple aspects of muscle metabolism and physiology? Actinin-2 and actinin-3 serve as a scaffold to anchor many signaling proteins and metabolic enzymes to the Z-disk [63]. Most of these interactions are probably shared by both muscle actinin isoforms, though this has often not been explicitly tested, nor have actual binding affinities been compared. Assuming there are some differential interactions of signaling proteins with actinin-2 versus actinin-3, then alterations in sarcomeric signal transduction in humans or mice lacking actinin-3 could drive a program of gene expression resulting in the actinin-3 null muscle phenotype. Calsarcin-2 (CALS-2, Myozenin-1, FATZ), a regulator of calcineurin signaling, displays just such a differential interaction [64]. Actinin-3 deficient muscle in both mice and humans show enhanced calcineurin signaling, probably as a result of increased binding of calsarcin-2 to actinin-2 in the absence of actinin-3 [64]. Calcineurin signaling is known to shift muscle fibers towards an oxidative phenotype [65–67], providing a potential mechanism to explain the changes in muscle metabolism in actinin-3 knockout mice [64]. Whether alterations in other signaling pathways are also involved remains to be seen.

### Actinin-4

Actinin-4 is regarded as a non-muscle isoform that is expressed widely, in a pattern that largely overlaps actinin-1 expression. These two isoforms show a high degree of sequence homology and have similar actin binding properties [15]. Three significant functional differences between actinin-1 and actinin-4 are apparent however. Firstly, aberrant actinin-4 expression has been described in many tumor types and has been linked to infiltrative phenotypes and poor outcomes in several cancers [68–75]. By contrast, over-expression of actinin-1 in tumor tissues has not been widely reported. Secondly, human studies have identified five dominant *ACTN4* mutations that cause the kidney disease focal segmental glomerulosclerosis (FSGS) [76, 77] (Table 1), while actinin-4 knockout mice exhibit altered podocyte morphology, develop glomerular disease, and finally, experience kidney failure [31]. In humans a kidney-specific role for actinin-4 can be simply explained by the lack of actinin-1 expression in the kidney [76], however in mice both actinin-1 and −4 are expressed in podocytes, yet actinin-1 cannot compensate for a loss of actinin-4 [31]. Thirdly, actinin-4 can translocate to the nucleus and play a role in transcriptional regulation [78–84]. This ability may be shared by actinin-2 [85], but a similar, “non-canonical” role for actinin-1 has not been reported. The diverse cell biological roles of actinin-1 and actinin-4 have recently been reviewed [30], and the roles of actinin-4 in cancer and kidney physiology are described in detail in other reviews in this special issue [86, 87]. For these reasons actinin-4 is not discussed further here.

## Conclusions

The *ACTN* gene family presents a fascinating case study in genetics. Through the course of evolution, from their origin in early eukaryotes, actinins have performed a core function as actin cross-linking proteins in cells. As more complex multi-cellular metazoan species evolved, actinin’s cross-linking activity was required in an increasing number of cell biological contexts in different cell and tissue types. Alternative splicing in the ABD and CaM domains arose, most likely to tweak actin-binding properties for tissue-specific functions. In vertebrates, gene duplications facilitated sub-functionalization compared to the ancestral invertebrate actinin, as well as neo-functionalization. In mammals, actinin-1 probably retained the broadest range of ancestral protein functions. Sub-functionalization saw actinin-2 specialize as the major thin filament cross-linker of the sarcomeric Z-disk in heart and skeletal muscle, as well as playing a role at neuronal synapses. Actinin-3 assumed an even more specific role in fast muscle fibers, where it is required for maintenance of the anaerobic metabolic phenotype of these fibers. Actinin-4 has largely overlapping expression and functions with actinin-1, except in the kidney. It has acquired, probably through neo-functionalization, a role in the cell nucleus as a transcriptional regulator and a unique ability to promote an aggressive cancer phenotype when over-expressed. Missense mutations in *ACTN1*, *ACTN2* and ACTN4 cause dominantly inherited platelet, cardiac and renal disorders respectively, while a nonsense mutation in *ACTN3* seems to have been beneficial during the recent evolution of some human populations. Still our understanding of molecular mechanisms linking these mutations to their respective phenotypes is incomplete and represents an ongoing challenge to researchers in this field.

## Additional file

Additional file 1:
**Multiple sequence alignment of selected actinin protein sequences.** An alignment prepared using ClustalX of human actinin-1, −2, −3 and −4, as well as actinin sequences from select model organisms. Sequence conservation is plotted beneath the alignment and conserved residues marked and color-coded according to the default CustalX settings. Locations of PFAM protein domains as identified in the Protein Data Bank for human actinin-2 (PDB ID: 4D1E) are indicated in red below the alignment. Known disease/phenotype associated mutations for the human actinins are identified in red text above the alignment. Accession numbers for sequences are as follows: *Hs*ACTN1 = NP_001093.1; *Hs*ACTN2 = NP_001094.1; *Hs*ACTN3 = NP_001095.2; *Hs*ACTN4 = NP_004915.2; *Dd*ACTN = XP_646979; *DmACTN* = NP_477484; *Ce*ACTN = NP_506127; *Sp*ACTN = NP_594295. Hs *= Homo sapiens;* Dm *= Drosophila melanogaster;* Ce *= Caenorhabditis elegans;* Dd *= Dictyostelium discoideum;* Sp *= Schizosaccharomyces pombe*. (PDF 3345 kb*)*

## Abbreviations

CH: Calponin homology
ABD: Actin-binding domain
SLR: Spectrin-like repeats
CaM: Calmodulin-like
CNS: Central nervous system
CMTP: Congenital macrothrombocytopenia
HCM: Hypertrophic cardiomyopathy
DCM: Dilated cardiomyopathy
FSGS: Focal segmental glomerulosclerosis
