# Supplementary material for: The actinin family of actin cross-linking proteins – a genetic perspective
Source: Cell Biosci. 2015 Aug 25;5:49. doi: 10.1186/s13578-015-0029-7 (PMC4550062; doi:10.1186/s13578-015-0029-7)
Supplement: Additional file 1: — Multiple sequence alignment of selected actinin protein sequences. An alignment prepared using ClustalX of human actinin-1, −2, −3 and −4, as well as actinin sequences from select model organisms. Sequence conservation is plotted beneath the alignment and conserved residues marked and color-coded according to the default CustalX settings. Locations of PFAM protein domains as identified in the Protein Data Bank for human actinin-2 (PDB ID: 4D1E) are indicated in red below the alignment. Known disease/phenotype associated mutations for the human actinins are identified in red text above the alignment. Accession numbers for sequences are as follows: HsACTN1 = NP_001093.1; HsACTN2 = NP_001094.1; HsACTN3 = NP_001095.2; HsACTN4 = NP_004915.2; DdACTN = XP_646979; DmACTN = NP_477484; CeACTN = NP_506127; SpACTN = NP_594295. Hs = Homo sapiens; Dm = Drosophila melanogaster; Ce = Caenorhabditis elegans; Dd = Dictyostelium discoideum; Sp = Schizosaccharomyces pombe. (PDF 3345 kb) [file 13578_2015_29_MOESM1_ESM.pdf]

Additional file 1

Multiple sequence alignment of selected actinin protein sequences

An alignment prepared using ClustalX of human actinin-1, -2, -3 and -4, as well as actinin sequences from select model organisms. Sequence conservation is plotted beneath the alignment and conserved residues marked and color-coded according to the default CustalX settings. Locations of PFAM protein domains as identified in the Protein Data Bank for human actinin-2 (PDB ID: 4D1E) are indicated in red below the alignment. Known disease/phenotype associated mutations for the human actinins are identified in red text above the alignment. Accession numbers for sequences are as follows: *Hs*ACTN1 = NP_001093.1; *Hs*ACTN2 = NP_001094.1; *Hs*ACTN3 = NP_001095.2; *Hs*ACTN4 = NP_004915.2; *Dd*ACTN = XP_646979; *DmACTN* = NP_477484; *Ce*ACTN = NP_506127; *Sp*ACTN = NP_594295. Hs *= Homo sapiens;* Dm *= Drosophila melanogaster;* Ce *= Caenorhabditis elegans;* Dd *= Dictyostelium discoideum;* Sp *= Schizosaccharomyces pombe*

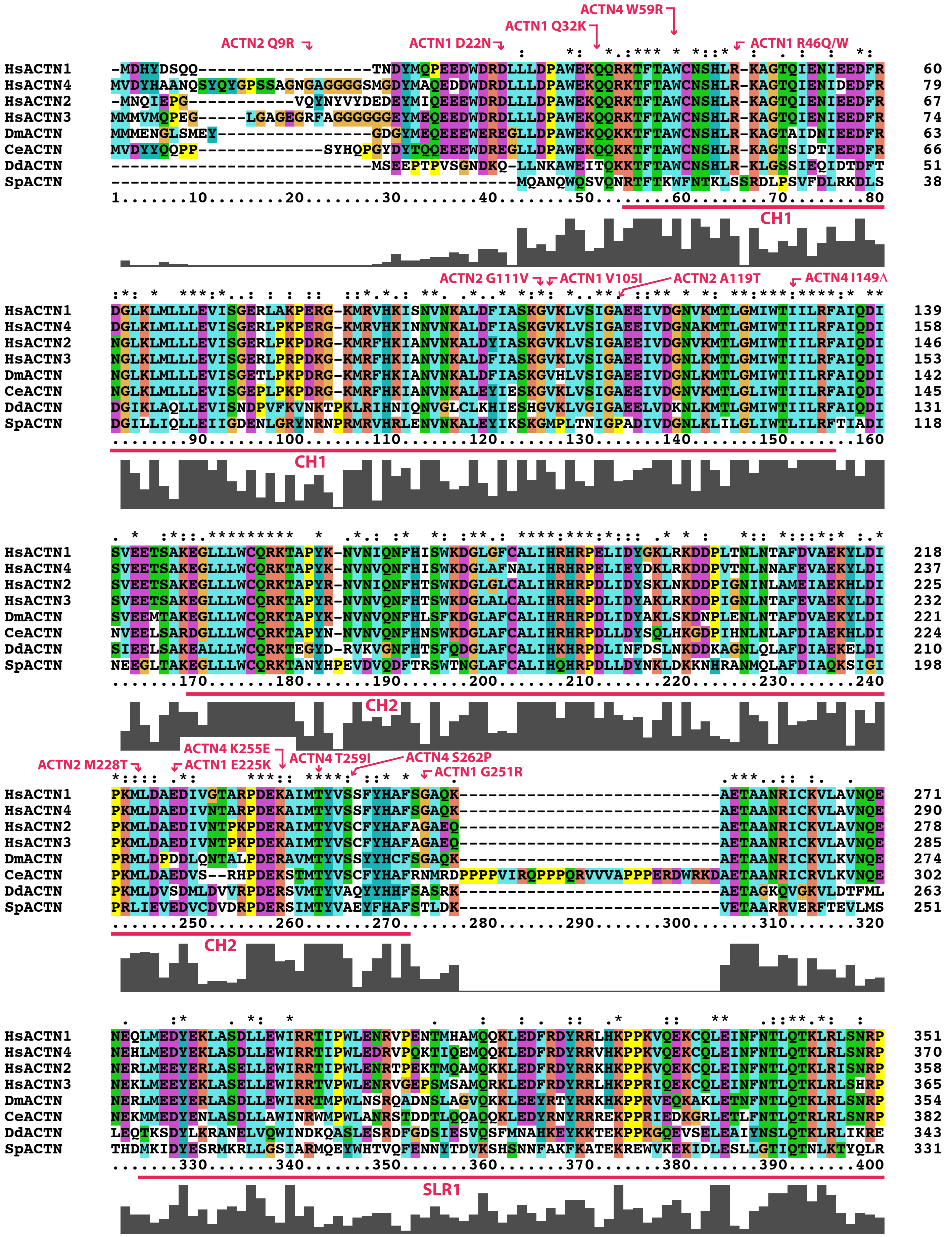
ACTN2 Q9R
ACTN1 D22N
ACTN1 Q32K
ACTN4 W59R
ACTN1 R46Q/W
HsACTN1 -MDHYDSQQ------------------TNDYMQPEEDWDRDLLLDPAWEKQQRKTFTAWCNSHLR-KAGTQIENIEEDFR 60
HsACTN4 MVDYHAANQSYQYGPSSAGNGAGGGGSMGDYMAQEDDWDRDLLLDPAWEKQQRKTFTAWCNSHLR-KAGTQIENIDEDFR 79
HsACTN2 -MNQIEPG-----------VQYNYVYDEDEYMIQEEEWDRDLLLDPAWEKQQRKTFTAWCNSHLR-KAGTQIENIEEDFR 67
HsACTN3 MMMVMQPEG-----LGAGEGRFAGGGGGGEYMEQEEDWDRDLLLDPAWEKQQRKTFTAWCNSHLR-KAGTQIENIEEDFR 74
DmACTN MMMENGLSMEY----------------GDGYMEQEEEWEREGLLDPAWEKQQKKTFTAWCNSHLR-KAGTAIDNIEEDFR 63
CeACTN MVDYYQQPP-------------SYHQPGYDYTQQEEEWDREGLLDPAWEKQQKKTFTAWCNSHLR-KAGTSIDTIEEDFR 66
DdACTN ---------------------------MSEEPTPVSGNDKQ-LLNKAWEITQKKTFTAWCNSHLR-KLGSSIEQIDTDFT 51
SpACTN --MQANQWQSVQNRTFTKWFNTKLSSRDLPSVFDLRKDLS 38
1.......10.......20.......30.......40.......50.......60.......70.......80
CH1
ACTN2 G111V
ACTN1 V105I
ACTN2 A119T
ACTN4 I149Δ
HsACTN1 DGLKLMLLLEVISGERLAKPERG-KMRVHKISNVNKALDFIASKGVKLVSIGAEEIVDGNVKMTLGMIWTIILRFAIQDI 139
HsACTN4 DGLKLMLLLEVISGERLPKPERG-KMRVHKINNVNKALDFIASKGVKLVSIGAEEIVDGNAKMTLGMIWTIILRFAIQDI 158
HsACTN2 NGLKLMLLLEVISGERLPKPDRG-KMRFHKIANVNKALDYIASKGVKLVSIGAEEIVDGNVKMTLGMIWTIILRFAIQDI 146
HsACTN3 NGLKLMLLLEVISGERLPRPDKG-KMRFHKIANVNKALDFIASKGVKLVSIGAEEIVDGNLKMTLGMIWTIILRFAIQDI 153
DmACTN NGLKLMLLLEVISGETLPKPDRG-KMRFHKIANVNKALDFIASKGVHLVSIGAEEIVDGNLKMTLGMIWTIILRFAIQDI 142
CeACTN NGLKLMLLLEVISGEPLPKPDRG-KMRFHKIANVNKALEYIESKGVKLVSIGAEEIVDGNVKMTLGLIWTIILRFAIQDI 145
DdACTN DGIKLAQLLEVISNDPVFKVNKTPKLRIHNIQNVGLCLKHIESHGVKLVGIGAEELVDKNLKMTLGMIWTIILRFAIQDI 131
SpACTN DGILLIQLLEIIGDENLGRYNRNPRMRVHRLENVNKALEYIKSKGMPLTNIGPADIVDGNLKLILGLIWTLILRFTIADI 118
........90.......100.......110.......120.......130.......140.......150.......160
CH1
HsACTN1 SVEETSAKEGLLLWCQRKTAPYK-NVNIQNFHISWKDGLGFCALIHRHRPELIDYGKLRKDDPLTNLNTAFDVAEKYLDI 218
HsACTN4 SVEETSAKEGLLLWCQRKTAPYK-NVNVQNFHISWKDGLAFNALIHRHRPELIEYDKLRKDDPVTNLNNAFEVAEKYLDI 237
HsACTN2 SVEETSAKEGLLLWCQRKTAPYR-NVNIQNFHTSWKDGLGLCALIHRHRPDLIDYSKLNKDDPIGNINLAMEIAEKHLDI 225
HsACTN3 SVEETSAKEGLLLWCQRKTAPYR-NVNVQNFHTSWKDGLALCALIHRHRPDLIDYAKLRKDDPIGNLNTAFEVAEKYLDI 232
DmACTN SVEEMTAKEGLLLWCQRKTAPYK-NVNVQNFHLSFKDGLAFCALIHRHRPDLIDYAKLSKDNPLENLNTAFDVAEKYLDI 221
CeACTN NVEELSARDGLLLWCQRKTAPYN-NVNVQNFHNSWKDGLAFCALIHRHRPDLLDYSQLHKGDPIHNLNLAFDIAEKHLDI 224
DdACTN SIEELSAKEALLLWCQRKTEGYD-RVKVGNFHTSFQDGLAFCALIHKHRPDLINFDSLNKDDKAGNLQLAFDIAEKELDI 210
SpACTN NEEGLTAKEGLLLWCQRKTANYHPEVDVQDFTRSWTNGLAFCALIHQHRPDLLDYNKLDKKNHRANMQLAFDIAQKSIGI 198
.......170.......180.......190.......200.......210.......220.......230.......240
CH2
ACTN4 K255E
ACTN2 M228T
ACTN1 E225K
ACTN4 T259I
ACTN4 S262P
ACTN1 G251R
HsACTN1 PKMLDAEDIVGTARPDEKAIMTYVSSFYHAFSGAQK---------------------------AETAANRICKVLAVNQE 271
HsACTN4 PKMLDAEDIVNTARPDEKAIMTYVSSFYHAFSGAQK---------------------------AETAANRICKVLAVNQE 290
HsACTN2 PKMLDAEDIVNTPKPDERAIMTYVSCFYHAFAGAEQ---------------------------AETAANRICKVLAVNQE 278
HsACTN3 PKMLDAEDIVNTPKPDEKAIMTYVSCFYHAFAGAEQ---------------------------AETAANRICKVLAVNQE 285
DmACTN PRMLDPDDLQNTALPDERAVMTYVSSYYHCFSGAQK---------------------------AETAANRICKVLKVNQE 274
CeACTN PKMLDAEDVS--RHPDEKSTMTYVSCFYHAFRNMRDPPPPVIRQPPPQRVVVAPPPERDWRKDAETAANRICRVLKVNQE 302
DdACTN PKMLDVSDMLDVVRPDERSVMTYVAQYYHHFSASRK---------------------------AETAGKQVGKVLDTFML 263
SpACTN PRLIEVEDVCDVDRPDERSIMTYVAEYFHAFSTLDK---------------------------VETAARRVERFTEVLMS 251
.......250.......260.......270.......280.......290.......300.......310.......320
CH2
HsACTN1 NEQLMEDYEKLASDLLEWIRRTIPWLENRVPENTMHAMQQKLEDFRDYRRLHKPPKVQEKCQLEINFNTLQTKLRLSNRP 351
HsACTN4 NEHLMEDYEKLASDLLEWIRRTIPWLEDRVPQKTIQEMQQKLEDFRDYRRVHKPPKVQEKCQLEINFNTLQTKLRLSNRP 370
HsACTN2 NERLMEEYERLASELLEWIRRTIPWLENRTPEKTMQAMQKKLEDFRDYRRKHKPPKVQEKCQLEINFNTLQTKLRISNRP 358
HsACTN3 NEKLMEEYEKLASELLEWIRRTVPWLENRVGEPSMSAMQRKLEDFRDYRRLHKPPRIQEKCQLEINFNTLQTKLRLSHRP 365
DmACTN NERLMEEYERLASDLLEWIRRTMPWLNSRQADNSLAGVQKKLEEYRTYRRKHKPPRVEQKAKLETNFNTLQTKLRLSNRP 354
CeACTN NEKMMEDYENLASDLLAWINRWMPWLANRSTDDTLQQAQQKLEDYRNYRRREKPPRIEDKGRLETLFNTLQTRLRLSNRP 382
DdACTN LEQTKSDYLKRANELVQWINDKQASLESRDFGDSIESVQSFMNAHKEYKKTEKPPKGQEVSELEAIYNSLQTKLRLIKRE 343
SpACTN THDMKIDYESRMKRLLGSIARMQEYWHTVQFENNYTDVKSHSNNFAKFKATEKREWVKEKIDLESLLGTIQTNLKTYQLR 331
.......330.......340.......350.......360.......370.......380.......390.......400
SLR1

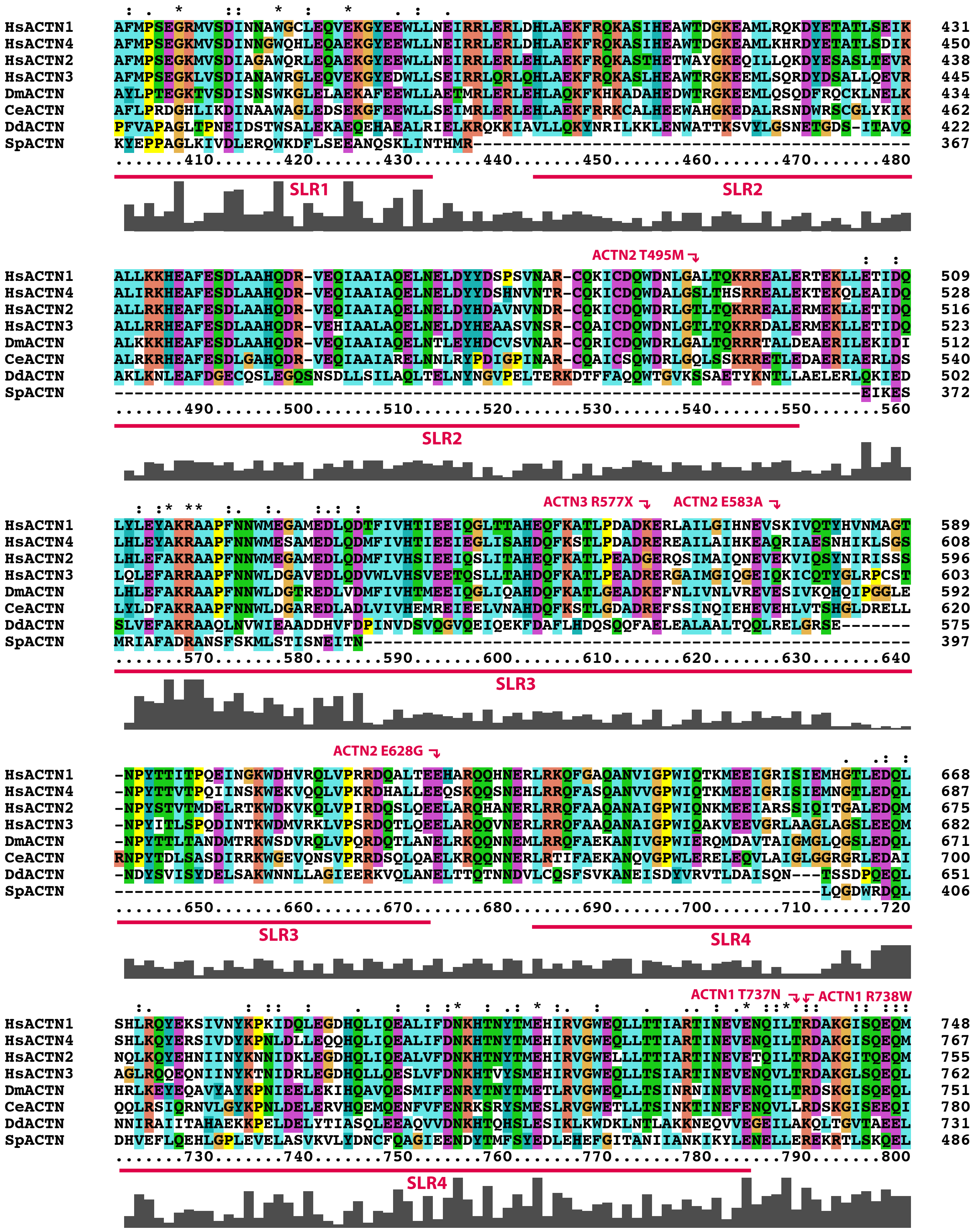
HsACTN1
HsACTN4
HsACTN2
HsACTN3
DmACTN
CeACTN
DdACTN
SpACTN
SLR1
SLR2
SLR3
SLR4
ACTN2 T495M
ACTN3 R577X
ACTN2 E583A
ACTN2 E628G
ACTN1 T737N
ACTN1 R738W

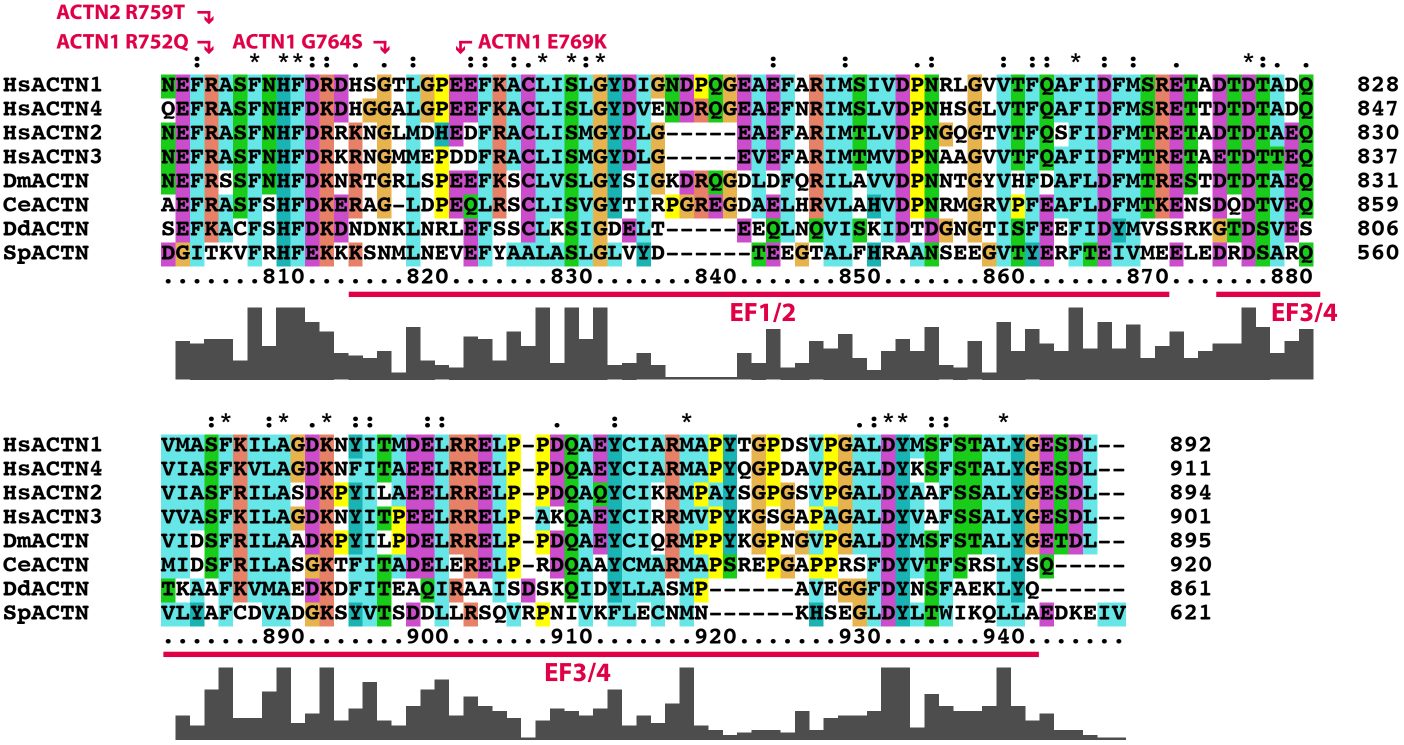
ACTN2 R759T
ACTN1 R752Q
ACTN1 G764S
ACTN1 E769K
HsACTN1 NEFRASFNHFDRDHSGTLGPEEFKACLISLGYDIGNDPQGEAEFARIMSIVDPNRLGVVTFQAFIDFMSRETADTDTADQ 828
HsACTN4 QEFRASFNHFDKDHGGALGPEEFKACLISLGYDVENDRQGEAEFNRIMSLVDPNHSGLVTFQAFIDFMSRETTDTDTADQ 847
HsACTN2 NEFRASFNHFDRRKNGLMDHEDFRACLISMGYDLG-----EAEFARIMTLVDPNGQGTVTFQSFIDFMTRETADTDTAEQ 830
HsACTN3 NEFRASFNHFDRKRNGMMEPDDFRACLISMGYDLG-----EVEFARIMTMVDPNAAGVVTFQAFIDFMTRETAETDTTEQ 837
DmACTN NEFRSSFNHFDKNRTGRLSPEEFKSCLVSLGYSIGKDRQGDLDFQRILAVVDPNNTGYVHFDAFLDFMTRESTDTDTAEQ 831
CeACTN AEFRASFSHFDKERAG-LDPEQLRSCLISVGYTIRPGREGDAELHRVLAHVDPNRMGRVPFEAFLDFMTKENSDQDTVEQ 859
DdACTN SEFKACFSHFDKDNDNKLNRLEFSSCLKSIGDELT-----EEQLNQVISKIDTDGNGTISFEEFIDYMVSSRKGTDSVES 806
SpACTN DGITKVFRHFEKKKSNMLNEVEFYAALASLGLVYD------TEEGTALFHRAANSEEGVTYERFTEIVMEELEDRDSARQ 560
.......810.......820.......830.......840.......850.......860.......870.......880
EF1/2
EF3/4
HsACTN1 VMASFKILAGDKNYITMDELRRELP-PDQAEYCIARMAPYTGPDSVPGALDYMSFSTALYGESDL-- 892
HsACTN4 VIASFKVLAGDKNFITAEELRRELP-PDQAEYCIARMAPYQGPDAVPGALDYKSFSTALYGESDL-- 911
HsACTN2 VIASFRILASDKPYILAEELRRELP-PDQAQYCIKRMPAYSGPGSVPGALDYAAFSSALYGESDL-- 894
HsACTN3 VVASFKILAGDKNYITPEELRRELP-AKQAEYCIRRMVPYKGSGAPAGALDYVAFSSALYGESDL-- 901
DmACTN VIDSFRILAADKPYILPDELRRELP-PDQAEYCIQRMPPYKGPNGVPGALDYMSFSTALYGETDL-- 895
CeACTN MIDSFRILASGKTFITADELERELP-RDQAAYCMARMAPSREPGAPPRSFDYVTFSRSLYSQ----- 920
DdACTN TKAAFKVMAEDKDFITEAQIRAAISDSKQIDYLLASMP------AVEGGFDYNSFAEKLYQ------ 861
SpACTN VLYAFCDVADGKSYVTSDDLLRSQVRPNIVKFLECNMN------KHSEGLDYLTWIKQLLAEDKEIV 621
.......890.......900.......910.......920.......930.......940.......
EF3/4
